# TGF-β signaling promotes eosinophil activation in inflammatory responses

**DOI:** 10.1038/s41419-024-07029-2

**Published:** 2024-08-30

**Authors:** Chen Zhu, Qingyu Weng, Shenwei Gao, Fei Li, Zhouyang Li, Yinfang Wu, Yanping Wu, Miao Li, Yun Zhao, Yinling Han, Weina Lu, Zhongnan Qin, Fangyi Yu, Jiafei Lou, Songmin Ying, Huahao Shen, Zhihua Chen, Wen Li

**Affiliations:** 1 Key Laboratory of Respiratory Disease of Zhejiang Province, Department of Respiratory and Critical Care Medicine, The Second Affiliated Hospital of Zhejiang University School of Medicine, Hangzhou, Zhejiang China; 2 Surgery Intensive Care Unit, The Second Affiliated Hospital of Zhejiang University School of Medicine, Hangzhou, Zhejiang China; 3 State Key Lab for Respiratory Diseases, Guangzhou, Guangdong China

**Keywords:** Inflammation, Asthma

## Abstract

Eosinophils, traditionally associated with allergic phenomena, play a pivotal role in inflammatory responses. Despite accumulating evidence suggesting their pro-inflammatory function upon activation, the underlying mechanisms governing eosinophil activation remain incompletely characterized. In this study, we investigate the local activation of pulmonary and colon eosinophils within the inflammatory microenvironment. Leveraging transcriptional sequencing, we identify TGF-β as a putative regulator of eosinophil activation, leading to the secretion of granule proteins, including peroxidase. Genetic deletion of TGF-β receptors on eosinophils resulted in the inhibition of peroxidase synthesis, affirming the significance of TGF-β signaling in eosinophil activation. Using models of HDM-induced asthma and DSS-induced colitis, we demonstrate the indispensability of TGF-β-driven eosinophil activation in both disease contexts. Notably, while TGF-β signaling did not significantly influence asthmatic inflammation, its knockout conferred protection against experimental colitis. This study delineates a distinct pattern of eosinophil activation within inflammatory responses, highlighting the pivotal role of TGF-β signaling in regulating eosinophil behavior. These findings deepen our comprehension of eosinophil-related pathophysiology and may pave the way for targeted therapeutic approaches in allergic and inflammatory diseases.

## Introduction

Eosinophils (Eos), since their discovery, have been regarded as a minor subset of immune cells during homeostasis [1]. As terminal effector cells, Eos are derived from bone marrow precursors and are distinguished by their abundant specific granules, containing cationic proteins such as eosinophil peroxidase (EPX), major basic protein (MBP), eosinophil cationic protein (ECP), and eosinophil-derived neurotoxin (EDN). These granules play a pivotal role in host defense, particularly during parasite infections. Eosinophils also hold significant responsibility in the context of allergic responses, as their local accumulation drives T helper 2 (Th2) immunity and prompts the release of related cytokines and chemokines. Notably, Th2-related inflammatory mediators, including interleukin (IL)-5, have demonstrated critical roles in enhancing eosinophilopoiesis and chemotaxis.

Eosinophil infiltration in inflamed airways and peripheral blood represents a hallmark of lung diseases, notably asthma, where Eos play a crucial role in the pathogenesis. James Lee and colleagues have demonstrated that Eos depletion effectively reduces airway inflammation, mucus secretion, and hypersensitivity in asthmatic mouse models [2]. Besides their significance in asthma, eosinophils also serve as vital immunoregulatory cells in various other diseases. Notably, the gut houses a substantial number of eosinophils that contribute to maintaining homeostasis [3]. Research has revealed their ability to suppress gut inflammation by modulating inefficient Th17 differentiation [4]. Additionally, eosinophil depletion accelerates DSS-induced colitis metabolically [5]. However, despite these insights, the precise mechanisms by which eosinophils influence inflammation in both asthma and colitis remain elusive.

Eosinophils undergo profound functional changes upon activation [6]. In the context of asthma, both airway and circulating eosinophils are activated and exhibit pro-inflammatory properties. Mesnil et al. reported that lung-resident eosinophils do not promote an inflammatory response during homeostasis [7]. In contrast, our previous study demonstrated eosinophil activation in asthmatic models and patients but not in endotoxin-induced pulmonary inflammation [8]. Upon activation, eosinophils exhibit diverse functional alterations, including the synthesis and release of secretory products such as granule proteins and other mediators, contributing to the pathogenesis of asthma in affected patients [9]. Notably, recent studies have linked post-activation eosinophil levels, particularly in bronchoalveolar lavage fluid (BALF), to the severity of asthma [10]. Hence, inhibiting eosinophil activation holds promise in alleviating asthma pathogenesis. In the intestinal environment, different phenotypic subsets of eosinophils have been identified [11]. However, concrete evidence implicating a particular subset as “activated” eosinophils remains elusive. Consequently, our understanding of the mechanisms governing eosinophil activation remains limited.

Transforming growth factor (TGF)-β constitutes a family of cytokines, and its elevated levels have been extensively reported in asthma [12]. TGF-β plays a crucial role in triggering the differentiation of Treg and Th17 cells and further modulates their activities. Of particular significance, TGF-β exerts a critical influence on the pathological processes in asthma, including airway remodeling, by activating fibroblasts. In our research, we demonstrate that TGF-β signaling promotes eosinophil activation in asthmatic mouse models. Moreover, elevated TGF-β levels have been observed in the DSS-induced colitis model, and the blockade of TGF-β effectively suppresses colitis pathogenesis.

The primary objective of the present research is to identify the key mediator responsible for eosinophil activation. Our comprehensive findings unequivocally demonstrate that TGF-β plays a pivotal role in promoting eosinophil activation across diverse inflammatory responses. Moreover, our study provides compelling evidence that inhibiting eosinophil activation holds the potential to mitigate pathological progression following inflammation.

## Materials and methods

### Mice

NJ.1638 mice and eoCre mice were generously provided by Prof. James Lee at Mayo Clinic, AZ. WT C57BL/6 mice were purchased from Shanghai SLAC laboratory animal Co., Ltd. Tgfbr2 flox/flox mice were generously gifted by Prof. Xinhua Feng at Life Sciences Institute, Zhejiang University. For all mouse strains utilized in our study, housing and breeding were conducted at the Laboratory Animal Center of Zhejiang University, Hangzhou, China.

The establishment of house-dust-mite-induced allergic airway models was in accordance with previous research [13]. Dextran sulfate-sodium salt (DSS) colitis models have been described in detail elsewhere [5]. In brief, colitis was induced by administration of dextran sodium sulfate (2.5–3.5% (w/v)) (d122354, Aladdin, China), to 10 weeks old mice for up to 6–8 days. Weight daily assessment and colon length after autopsy were measured as previously described.

All the mice were randomly allocated into control groups or models.

### Reagent

TGF-β and IL-5 were obtained from Suzhou Novoprotein Technology. House dust mites (HDM) were purchased from Greer Laboratories. DSS was procured from Shanghai Aladdin Biochemical Technology. Chitin and papain were acquired from Sigma Aldrich. The EPX antibody was generously provided by Prof. J.J. Lee. Additionally, p-Smad2, p-Smad3, and β-actin antibodies were purchased from Cell Signal Technology. The TGF-β ELISA kit was procured from Biolegend (Cat437707), and all experimental procedures were conducted following the instructions provided by the kit.

### Eosinophil isolation and culture from mice [8]

In NJ.1638 mice, circulating eosinophils were abundant. Briefly, peripheral blood from NJ.1638 mice was subjected to a Percoll (GE Healthcare) gradient separation. The resultant mixed cells, containing eosinophils along with other contaminant cells, were further purified using CD4^−^CD8^−^B220^−^Ter119^−^ magnetic beads isolation (Miltenyi Biotec). The purity of the isolated eosinophils exceeded 95% (Fig. S1).

For WT mice, bone-marrow-derived non-adherent mononuclear cells (NAMNCs) were seeded at 1 × 10^6^/ml in IMDM completed medium containing IMDM (Iscove’s modified Dulbecco’s medium; Invitrogen) with 20% heat-inactivated FBS (Gibco), 100 IU/ml penicillin and 10 mg/ml streptomycin (Sigma-Aldrich), 2mM l-glutamine(Sigma-Aldrich), 1× nonessential amino acids (Sigma-Aldrich), 1 mM sodium pyruvate (Sigma-Aldrich) and 0.006‰ β-mercaptoethanol (Sigma-Aldrich). 100 ng/ml rmFlt-3L (Novoprotein) and 100 ng/ml rmSCF (Novoprotein) were added from day 0 to day 4. On day 4 and day 8, the medium was changed to contain 10 ng/ml rmIL-5 (Novoprotein) and was devoid of rmFlt-3L and rmSCF. On day 10, the purity of the Eos was approximately 80–90%.

After purification, eosinophils were treated with 10 ng/ml TGF-β and 2 ng/ml IL-5 for 24 h. The viability of eosinophils was approximately 95%.

### Flow cytometry

Mice were euthanized by pentobarbital anesthesia, and the lungs were subsequently flushed with 10 ml of PBS through right ventricular injection to maximize the removal of trapping leukocytes in circulation. Afterward, bilateral lungs were harvested, cut into pieces, and then digested with 1 mg/ml collagenase I (Sigma) for 90 min. The digested tissues were finally filtered to obtain a single-cell suspension for further analysis.

Flow cytometric analysis (FACS) was performed using Cytoflex (Beckman Coulter), and all the results were analyzed using Flowjo X software (Treestar). The fluorescence-conjugated antibodies utilized in the analysis are listed in Table 1. Isotype control antibodies corresponding to each fluorescence-conjugated antibody were purchased from Biolegend and employed for staining in flow cytometry analysis. Representative FACS gating strategies are represented as Fig. S2.

**Table 1:** FACS antibodies.

Flow cytometry antibody	Manufacturer
Anti-mouse CD45-FITC	Biolegend
Anti-mouse CD45-PB450	Biolegend
Anti-mouse CD11c-APC	Biolegend
Anti-mouse SiglecF-PE	Biolegend
Anti-mouse Ly6G-FITC	Biolegend
Anti-mouse CD101-PEcy7	Biolegend
Anti-human CD45-FITC	Biolegend
Anti-human Siglec-8-APC	Biolegend
Anti-human CCR3-BV510	Biolegend
Anti-human CD101-PE	Biolegend

### Immunofluorescence

Eosinophils were collected by centrifugation at 800 × *g* for 5 min. Subsequently, cells were fixed with a 4% formaldehyde solution for 15 min, permeabilized with 0.5% TritonX-100 for 20 min, and blocked by incubation with 3% BSA in PBS for at least 1 h. The cells were then incubated with the EPX antibody at 4 °C for 12 h. After washing, the cells were further incubated with fluorescent antibodies conjugated to Alexa Fluor 488 or 555 (Life Technologies). Cell nuclei were stained with DAPI. Imaging was performed using an automated Nikon Eclipse Ni microscope with Nikon’s Elements software (Nikon Instruments). For each sample, at least 300 cells were imaged and analyzed from random views.

### Eosinophil peroxidase enzymatic activity assay

Eosinophil peroxidase activity was assessed by microtitration with a final volume of 125 µL. Each well comprised 75 µL of the OPD matrix solution (50 mM Tris-HCl, pH 8, 0.1% Triton X-100 (Sigma Aldrich), 8.8 mM H_2_O_2_ (Sigma Aldrich), 6 mM potassium bromide (KBr, Sigma Aldrich), and 10 mM o-phenylenediamine (OPD, Sigma Aldrich)), along with 50 µL of the sample. Furthermore, 10 mM of the peroxidase inhibitor resorcinol (1,3-benzenediol, Sigma-Aldrich) was included as a negative control. The system was thoroughly mixed for analysis and subsequently incubated at 37 °C for 30 min. Following incubation, 50 μL of 2 N H_2_SO_4_ was added to stop the reaction, and the absorbance of each sample was measured at 490 nm using a Quant microplate spectrophotometer. The relative enzyme activity was determined based on the measurements obtained.

### Eosinophil transwell migration assay [14]

In brief, CD101^−^ and CD101^+^ eosinophils were sorted from HDM mice. Eosinophils at a concentration of 10^5^/mL were seeded in a 1:1 ratio in the upper chamber of a transwell system, with eotaxin (20 ng/mL, Peprotech) added to the medium in the lower chamber. After 30 min of incubation at 37 °C, eosinophils in the bottom chamber were counted, and subsets were analyzed by FACS. The migration rate was determined by the ratio of eosinophils in the bottom chamber to the total number of seeded eosinophils. Detailed methods are provided in the Materials and Methods section of the revised manuscript.

### Electron microscopy

Eosinophils were extracted and collected from the peripheral blood of NJ.1638 mice. The collected cells were fixed in 2.5% glutaraldehyde, followed by 4% osmium tetroxide. Dehydration of the fixed cells was performed using graded ethanol, and subsequently, the cells were stained with uranyl acetate and lead Reynolds citrate. Slices from each block were then mounted on separate electron microscope grids for examination using transmission electron microscopy. During the examination, the operator systematically scanned the entire grid from left to right and top to bottom, photographing several eosinophils and all randomly selected particles present in the section.

### Lung histology

EPX immunohistochemistry staining was conducted to detect eosinophils. Additionally, H&E staining was performed to evaluate inflammation, while PAS staining was utilized to examine mucus secretion. The stained sections were visualized using an Olympus BX51 microscope equipped with a 4/0.3 NA objective and DP70 digital camera. The inflammatory score of both HDM [15] and DSS [16] model was calculated as previously described elsewhere.

### RNA isolation and quantitative real-time PCR analysis

Eosinophils or lung tissues were lysed using RNAiso reagent (Takara, Japan) following the manufacturer’s protocol. The primers required for real-time PCR were obtained from Shanghai Bioengineering (Shanghai, China). For reverse transcription and real-time PCR, the PrimeScript TM RT-PCR kit (Takara) and SYBR Primix TaqTM (Takara) were utilized, respectively. The specific primers used in the experiments are listed below: Actb: forward, 5′-AGAGGGAAATCGTGCGRGAC-3′; reverse, 5′-CAATAGTGACCTGGCCGT-3′. Epx: forward, 5′-CTCACCCAACACGCTGAAG-3′; reverse, 5′-TTTTCTGTGTGTGATTGTAGGCA-3′. Il4: forward, 5′- GGTCTCAACCCCCAGCTAGT-3′; reverse, 5′-GCCGATGATCTCTCTCAAGTGAT-3′. Il13: forward, 5′-CAGCCTCCCCGATACCAAAAT-3′; reverse, 5′-GCGAAACAGTTGCTTTGTGTAG-3′. Muc5ac: forward, 5′-CTGTGACATTATCCCATAAGCCC-3′; reverse, 5′- AAGGGGTATAGCTGGCCTGA-3′. Il1b: forward, 5′-CCTCCTTGCCTCTGATGG-3′; reverse, 5′-AGTGCTGCCTAATGTCCC-3′. Il6: forward, 5′-CTGCAAGAGACTTCCATCCAG-3′; reverse, 5′-AGTGGTATAGACAGGTCTGTTGG-3′. Il10: forward, 5′-GCTCTTACTGACTGGCATGAG-3′; reverse, 5′-CGCAGCTCTAGGAGCATGTG-3′.

The relative abundance of certain targets was analyzed in the delta-delta CT method and normalized to Actb.

### Statistics analysis

All data were analyzed with Prism 8.0 (GraphPad Software). The nonparametric Mann–Whitney test was used for all statistical comparisons. All data were represented as Mean ± SD. *p < 0.05; **p < 0.01; ***p < 0.001; ****p < 0.0001.

## Results

### Increased infiltration of locally activated Eos in various inflammatory responses

**Fig. 1:**
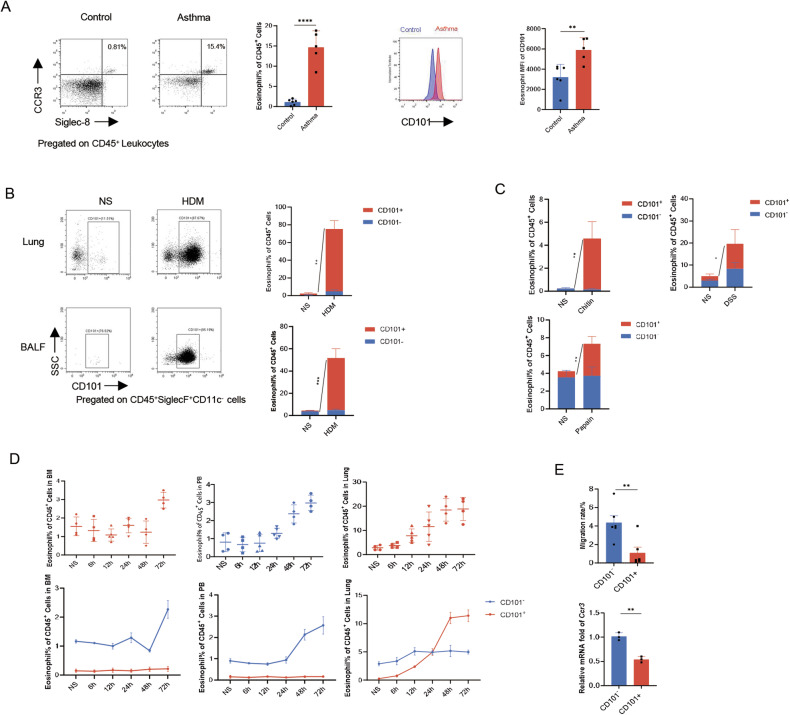
Increased infiltration of locally activated Eos in various inflammatory responses. **A** The proportion of Eos in peripheral blood of asthmatic patients, and CD101 expression (as mean fluorescence intensity) in circulating Eos in asthmatic patients. **B** Percentage of Eos subsets in inflammatory cells in BALF and lung homogenates. BALF cells were harvested 24 h after last exposure in HDM-induced mouse model. Eos were labeled as live CD45^+^SiglecF^+^CD11c^−^ cells. *n* = 4. **C** Percentage of Eos subsets in lung homogenates in chitin and papain induced mouse models. *n* = 4. **D** Dynamic changes (6 h, 12 h, 24 h, 48 h, 72 h from last exposure) of Eos in bone marrow, peripheral blood and lung tissue after last exposure in HDM model. **E** Migrated rate of Eos in transwell migration assay, and Ccr3 mRNA level of different Eos subsets. Sample size is indicated as individual plots in column graphs. Data are triplicate by individual experiments except A. **p < 0.01; ***p < 0.001; ****p < 0.0001.

Activated eosinophils have been observed to infiltrate the inflammatory microenvironment of the asthmatic lung. Previous studies have highlighted distinct functions of activated eosinophils compared to steady-state eosinophils resident in the lung, commonly referred to as regulatory eosinophils [7]. CD101 has been identified as one of the surface markers to distinguish activated eosinophils from steady-state eosinophils, with activated eosinophils labeled as CD101^+^ [7, 8]. To validate this observation, we initially compared the distribution of different eosinophil subsets in the peripheral blood of asthmatic patients. The results indicated an increased number of eosinophils in asthmatic patients, with a predominant proportion expressing CD101hi (Fig. 1A). Furthermore, in a house dust mite (HDM)-induced allergic airway inflammation mouse model, eosinophils, particularly CD101^+^ eosinophils, were found to increase in both lung tissue and bronchoalveolar lavage fluid (BALF) (Fig. 1B). These findings demonstrated that eosinophils, especially the CD101^+^ subset, are elevated in the context of asthma. To investigate whether CD101^+^ eosinophils are universally involved in pulmonary inflammatory responses, we analyzed eosinophils in other eosinophil-related pulmonary disease models induced by chitin and papain. Similarly, activated CD101^+^ eosinophils increased during pulmonary inflammation in each of these models (Fig. 1C). Notably, even in the DSS-induced experimental colitis model, the population of CD101^+^ eosinophils were augmented (Fig. 1C). Collectively, these results indicate a generalized increase in activated eosinophils in various inflammatory settings.

As previous research has indicated, pulmonary eosinophil infiltration is contingent upon their development in the bone marrow, followed by recruitment from the peripheral blood in asthma [17]. In a steady state, the majority of eosinophils exist in an inactivated state. To explore the origin of activated eosinophils in the asthmatic lungs, we monitored the dynamic changes of CD101^+^ eosinophils in the bone marrow, peripheral blood, and lung tissues of asthmatic mice. Our data revealed that within the first 24 h after house dust mite (HDM) exposure, eosinophils gradually increased in all the bone marrow, peripheral blood, and lung tissues, and the population of eosinophils was predominantly CD101^−^. Subsequently, at 24 h post inhalation, the levels of CD101^+^ eosinophils increased rapidly, becoming comparable to those of CD101^−^ in the lungs (Fig. 1D). Following this, pulmonary CD101^+^ eosinophils steadily accelerated in numbers, eventually surpassing CD101^−^ eosinophils, while CD101^−^ eosinophils remained predominant in the bone marrow and peripheral blood. Based on these findings, we hypothesized that eosinophils were locally activated in the asthmatic microenvironment after their recruitment from the bone marrow or blood.

In line with previous studies, eosinophil migration is dependent on a specific chemokine, known as eotaxin [17]. Typically, eotaxin drives eosinophil recruitment by binding to its receptor CCR3. To further substantiate the local activation of eosinophils, we conducted migration experiments with CD101^−^ and CD101^+^ eosinophils from asthmatic mice. In this migration system, eosinophils were seeded inside the transwell, and eotaxin was added to the medium outside the transwell. The results demonstrated that the proportion of CD101^−^ eosinophils migrating into the medium exceeded that of CD101^+^ eosinophils. Furthermore, the abundance of *Ccr3* was higher in CD101^−^ eosinophils compared to CD101^+^ eosinophils (Fig. 1E). These data collectively suggested that CD101^+^ eosinophils exhibited lower recruitment potential. In conclusion, our findings support the notion that eosinophils migrate to the lungs and subsequently undergo local activation within the asthmatic inflammatory milieu.

### TGF-β signaling pathway promoted Eos activation

**Fig. 2:**
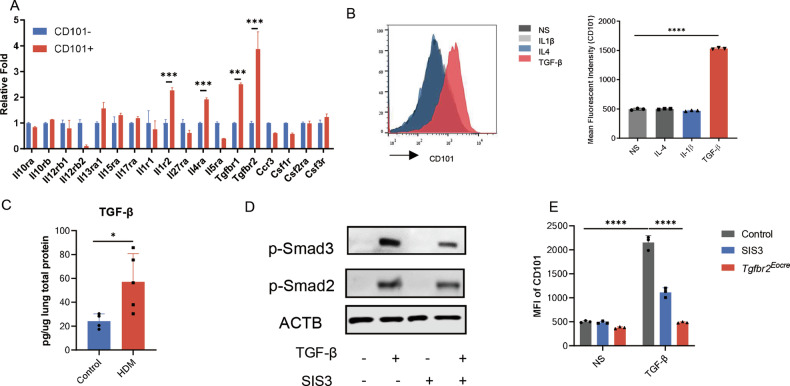
TGF-β signaling pathway promoted Eos activation. **A** Differential expression of receptors of CD101−/+ eosinophils according to the result of RNAseq. *n* = 3. **B** The expression of CD101 in stimulated Eos from NJ.1638 mice. Left, representative image of flow cytometry. Right, mean fluorescence intensity of Eos treated with 10 ng/ml IL-4, 20 ng/ml IL-1β and 10 ng/ml TGF-β. **C** TGF-β concentration in lung homogenates of HDM model. **D** Expression of Smads signaling pathway in Eos after TGF-β stimulation. **E** Mean fluorescence intensity of CD101 in Eos. Eos were treated with 5 μM SIS3 intervention, or purified from NJ.1638×Eocre-*Tgfbr2* ^*f/f*^ mouse (which was hybrid by NJ.1638 and Eocre-*Tgfbr2* ^*f/f*^ mouse, an Eos abundant and Tgfbr2 specific knockout in Eos mouse strain). Sample size is indicated as individual plots in column graphs. Data are triplicated by individual experiments except A. *p < 0.05; ***p < 0.001; ****p < 0.0001.

Eosinophils play a pivotal role in asthmatic inflammation, motivating us to delve into the mechanisms underlying their activation in asthma. Building upon our previous study [8], we conducted transcriptome sequencing of CD101^−^ and CD101^+^ eosinophils obtained from asthmatic mice. In our exploration of potential receptors, we observed increased relative expression of *Il1r1*, *Il4ra*, and *Tgfbr1* in activated eosinophils, while other receptors showed no significant changes (Fig. 2A). Drawing from the RNAseq results, we postulated that the corresponding ligands might be responsible for eosinophil activation. Consequently, eosinophils were subjected to individual treatments with IL-1β, IL-4, and TGF-β, and the expression of CD101 was analyzed in vitro. Following 24 h of exposure, we discovered that only TGF-β could induce the expression of CD101 in eosinophils (Fig. 2B). We also observed that the receptor for IL-12 (*Il12rb2*) was downregulated in CD101^+^ eosinophils; however, IL-12 did not induce the expression of CD101 in these cells (Fig. S3).

The involvement of TGF-β in asthma has been partially explored by researchers, with elevated TGF-β levels observed in both asthmatic patients and mice. As expected, the concentration of TGF-β in lung tissue was found to increase in asthmatic mice at an early stage (24 h, Fig. 2C). This finding suggests that TGF-β emanates from the asthmatic microenvironment, potentially contributing to eosinophil activation in mice.

The activation of eosinophils by TGF-β is well-established, involving the canonical Smads pathway upon binding to the TGF-β receptor (Tgfbr). To further investigate this classical pathway’s role in eosinophil activation, we examined whether TGF-β induced phosphorylation of Smad2 and Smad3 in eosinophils. Our findings provided preliminary evidence supporting that eosinophil activation is dependent on the TGF-β-Smads pathway (Fig. 2D). To reinforce this observation, we employed the Smads inhibitor SIS3 and utilized Tgfbr2 knockout mice (Eocre-Tgfbr2^flox/flox^). By treating eosinophils with TGF-β and subsequently assessing the expression of CD101 in the presence of these antagonists or genetic knockout, we obtained compelling results. As anticipated, both SIS3 and Tgfbr2 knockout effectively prevented eosinophil activation in vitro (Fig. 2E). Taken together, these results collectively underscore the significance of the TGF-β signaling pathway in eosinophil activation.

### TGF-β promoted Eos activation by synthesizing Eos peroxidase

In the context of asthmatic inflammation, eosinophils exhibit diverse and broad functions, acting as effector cells responsible for inducing tissue damage through the release of substantial amounts of products, including interleukin (IL-) 4, IL-13, and characteristic cationic proteins [9]. Intriguingly, our investigations revealed that TGF-β induced eosinophil activation without concurrently promoting IL-4 or IL-13 (Fig. S4). This observation prompted us to explore the impact of the TGF-β signaling pathway on eosinophil function.

**Fig. 3:**
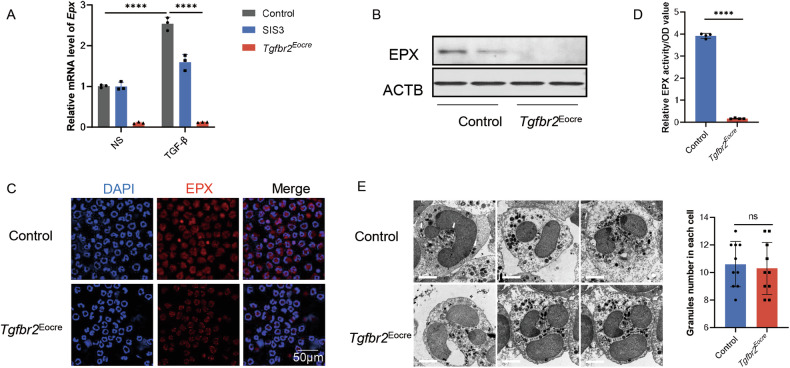
TGF-β promoted Eos activation by synthesizing Eos peroxidase. **A** Relative mRNA expression of *Epx* in Eos from NJ.1638×Eocre-*Tgfbr2*^*f/f*^ mouse. **B** Blots of EPX expression in Eos. **C** Immunofluorescence images of EPX expression in *Tgfbr2* knockout Eos. Scale bar, 50 μm. **D** Relative Eos peroxidase activity in *Tgfbr2* knockout Eos. **E** Left, Representative images on electron microscopy of Eos. Scale bar, 2 μm. Right, granules content in Eos. Sample size is indicated as individual plots in column graphs. Data are triplicate by individual experiments. ****p < 0.0001. ns not significant.

Based on our RNAseq data, we identified an increase in eosinophil peroxidase (Epx) upon activation. To validate our findings, we employed SIS3 and Eocre-Tgfbr2^flox/flox^ mice. Purified eosinophils from NJ.1638 mice were treated with TGF-β, and the results demonstrated that TGF-β induced a rise in *Epx* abundance in eosinophils, which was effectively inhibited by SIS3 (Fig. 3A). Similarly, the increase in *Epx* levels induced by TGF-β was also attenuated in eosinophils from Tgfbr2 knockout mice. Additionally, EPX synthesis during activation was impaired in Eocre-Tgfbr2^flox/flox^ mice, as confirmed by Western blot assay (Fig. 3B) and immunofluorescence (Fig. 3C). An assay for EPX activity further corroborated these findings, as the blockage of the TGF-β signaling pathway contributed to the decrease in EPX activity (Fig. 3D). In light of the decline in EPX secretion, we examined the granule secretion of all types, finding a similar number of granules in both Tgfbr2-null eosinophils and the control after activation, as observed through electron microscope analysis (Fig. 3E).

To provide further confirmation, bone marrow-derived eosinophils were cultured. After ten days, CD101 expression in eosinophils increased upon TGF-β administration, and inhibition of TGF-β prevented eosinophil activation (Fig. S5A). Furthermore, Tgfbr2 knockout mice exhibited reduced expression of eosinophil peroxidase during activation (Fig. S5B). These cumulative results indicate that eosinophil activation is characterized by increased EPX synthesis in response to TGF-β.

### Inhibition of the TGF-β signaling pathway prevented Eos activation but did not suppress asthmatic inflammation

**Fig. 4:**
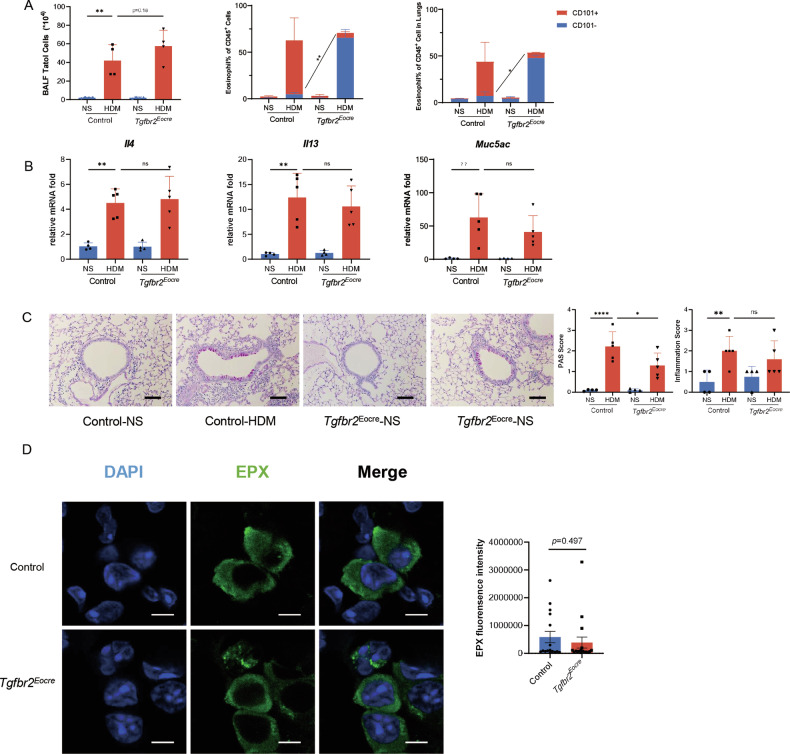
Inhibition of the TGF-β signaling pathway prevented Eos activation but did not suppress asthmatic inflammation. **A** Cellularity in HDM-induced asthma model. Left, total cells count in BALF. Middle, Eos subsets percentage in BALF cells. Right, Eos subsets percentage in lung homogenates. *n* = 4. **B** Relative mRNA expression of lung inflammatory cytokines *Il4, Il13* and *Muc5ac*. **C** Airway inflammation evaluation in asthmatic model. Left, representative images of lung histology. Scale bar, 200μm. Middle, PAS score of asthmatic mice. Right, inflammation score of asthmatic mice. **D** Immunofluorescence of lung tissue of HDM model. Left, representative image of immunofluorescence. EPX, green. DAPI, blue. Right, statistics of fluorescence intensity of EPX. Scale bar, 5 μm. Sample size is indicated as individual plots in column graphs. Data are triplicate by individual experiments. *p < 0.05; **p < 0.01; ****p < 0.0001. ns not significant.

To investigate the potential therapeutic implications of inhibiting eosinophil activation in asthma, we explored the impact of TGF-β inhibition on allergic airway inflammation in vivo. We established HDM-induced asthma models in both Eocre-Tgfbr2^flox/flox^ mice and their control counterparts (Tgfbr2^flox/flox^ mice) and assessed airway inflammation by analyzing bronchoalveolar lavage fluid (BALF) and lung homogenates. In accordance with our in vitro findings, TGF-β deficiency led to dysfunctional eosinophil activation in asthmatic mice, evident by a decrease in CD101^+^ eosinophils in both BALF and cell suspensions of lung tissue (Fig. 4A).

Surprisingly, however, despite the impairment of eosinophil activation in the Eocre-Tgfbr2^flox/flox^ mice, there was no significant difference in the total number of BALF cells or eosinophil percentage between the two groups (Fig. 4A). Similarly, in lung homogenates, no significant difference was observed in eosinophil quantity, regardless of TGF-β impairment or not. Regarding the Th2 response, we examined representative inflammatory markers in asthmatic mice, such as IL-4, IL-13, and IL-33. Strikingly, the levels of these inflammatory mediators showed no difference in Eocre-Tgfbr2^flox/flox^ mice (Fig. 4B).

Furthermore, when evaluating the pathology of asthmatic mice, we found no significant difference in terms of inflammatory infiltration or mucus production between the Eocre-Tgfbr2^flox/flox^ mice and their control group (Fig. 4C). In the experimental setting of immunofluorescence, EPX was visualized in green. In the HDM model (Fig. 4D), EPX exhibited diffused distribution surrounding granules, and the inhibition of TGF-β in eosinophils resulted in a comparable level of EPX. To further confirm these results, chronic asthmatic models were established and assessed at day 31 (Fig. S6A). Similar to the acute models, airway inflammation did not show any difference in either mouse strain (Fig. S6B).

In conclusion, TGF-β deficiency impaired eosinophil activation but did not significantly impact the overall asthmatic pathology.

### TGF-β signaling pathway blockade suppressed Eos activation and inflammation in colitis mice

**Fig. 5:**
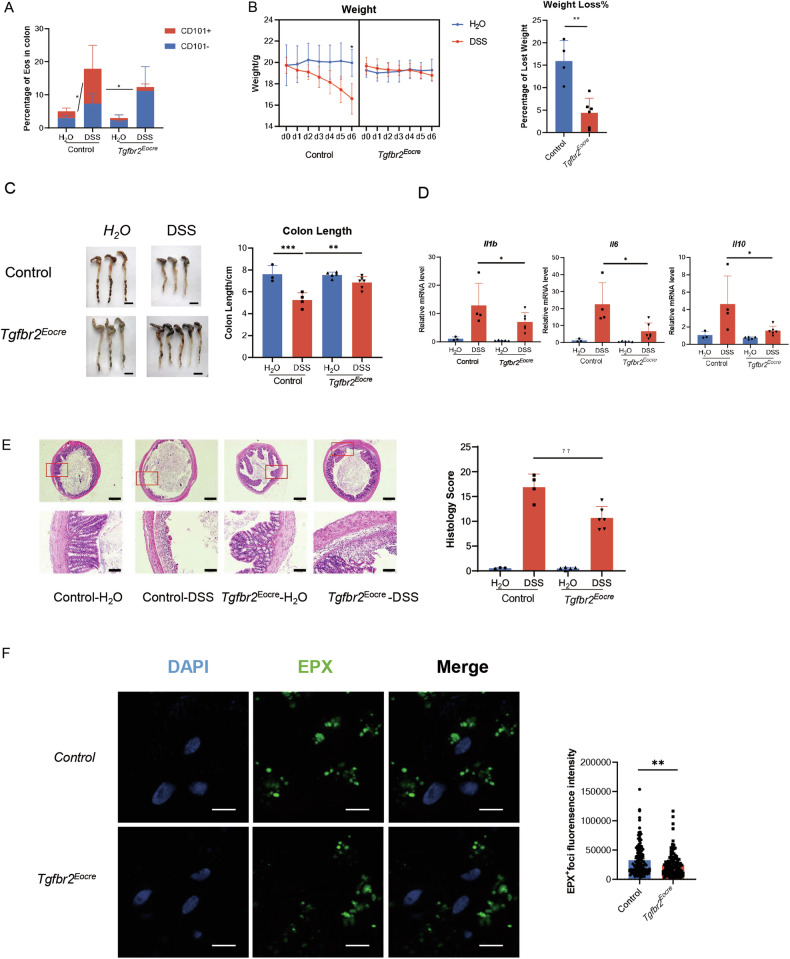
TGF-β signaling pathway blockade suppressed Eos activation and inflammation in colitis mice. **A** Different Eos subset percentage in colon of DSS-induced colitis mice. *n* = 3. **B** Weight loss in experimental colitis. Left, dynamic weight change of colitis mice after DSS exposure. n = 4. Right, percentage of weight loss after the establishment of DSS-induce colitis model. **C** Colon length shortening in experimental colitis. Left, representative images of mice colon. Scale bar, 1 cm. Right, Colon length of experimental colitis mice. **D** Relative mRNA expression of *IL-1β*, *IL-6* and *IL-10*. **E** Histology of experimental colitis. Left, representative images of colitis tissue. Right, inflammatory score of colitis. **F** Immunofluorescence of foci in colon tissue of DSS model. Left, representative image of immunofluorescence. EPX, green. DAPI, blue. Right, statistics of fluorescence intensity of EPX foci. Scale bar, 5 μm. Sample size is indicated as individual plots in column graphs. Data are triplicated by individual experiments. *p < 0.05; **p < 0.01; ***p < 0.001. ns not significant.

To further explore the role of eosinophil activation in colitis, we generated a DSS-induced colitis mouse model. In line with our previous findings in the HDM-induced asthma model, the proportion of CD101^+^ eosinophils increased in colitis mice, indicating the presence of activated CD101^+^ eosinophils in colitis, and confirming the role of TGF-β signaling in promoting eosinophil activation (Fig. 5A).

Colitis is characterized by pathological changes in the colon, mainly due to shortening caused by peri-colonic inflammatory infiltration, resulting in an overall decrease in body weight of the mice. After DSS administration, the body weight of colitis mice decreased, but this decline was reversed in Eocre-Tgfbr2 ^flox/flox^ mice, indicating a protective effect of TGF-β genetic knockout in preventing weight loss in colitis (Fig. 5B). Additionally, colon lengths were evaluated as a measure of colitis severity. The results revealed that colon lengths were shortened in the colitis model, but inhibition of TGF-β signaling protected the colon from shortening (Fig. 5C).

Furthermore, we examined inflammatory cytokines, such as IL-1β and IL-6, in colon tissue and found decreased levels in DSS-treated Eocre-Tgfbr2^flox/flox^ mice compared to Tgfbr2 ^flox/flox^ mice, indicating a reduction in inflammatory mediators due to TGF-β inhibition (Fig. 5D). Consistently, inflammatory infiltration in the peri-colon was alleviated in Tgfbr2 genetic knockout mice (Fig. 5E). Interestingly, EPX manifested as distinct foci, indicative of degranulation. The fluorescence intensity of EPX diminished in knockout mice, signifying a decreased level of degranulation following the inhibition of TGF-β in eosinophils (Fig. 5F). This observation aligns with our prior findings and serves to reinforce our conclusion.

In summary, our data demonstrate that TGF-β drives eosinophil activation, and inhibiting the TGF-β signaling pathway-induced eosinophil activation can ameliorate DSS-induced colitis. These findings suggest a potential therapeutic target for colitis by targeting eosinophil activation through TGF-β inhibition.

## Discussion

**Fig. 6:**
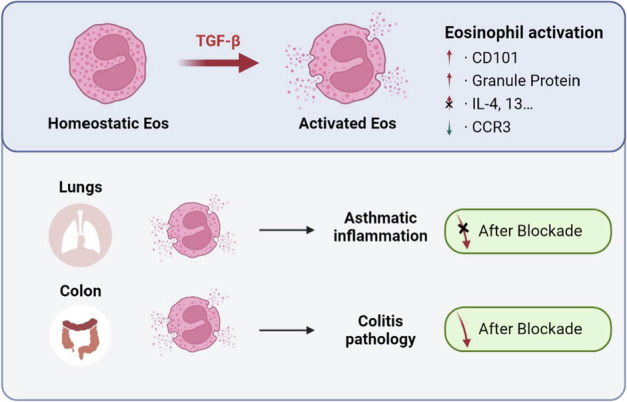
Summary. Our research suggested that TGF-β promote Eos local activation, represented as increased CD101 and granule protein. In asthma, blocking TGF-β induced Eos activation did not alleviate airway inflammation. But in colitis, blocking TGF-β could inhibit inflammatory response in colon.

In this study, we present evidence of eosinophil activation across various inflammatory responses, as summarized in Fig. 6. In asthma, TGF-β signaling was shown to activate eosinophils and induce peroxidase synthesis. However, inhibition of TGF-β signaling did not reduce airway inflammation in mice, likely due to the limited impact of eosinophil-derived peroxidase on asthmatic pathology. In contrast, in the DSS-induced colitis model, TGF-β enhanced eosinophil activation, and blocking the TGF-β receptor alleviated colitis inflammation. These results suggest that targeting the TGF-β signaling pathway could be a promising therapeutic strategy for eosinophil-associated inflammatory conditions, such as colitis. Nonetheless, the context-specific effects of eosinophil activation in different inflammatory disorders must be considered. Further research is needed to clarify the mechanisms involved and explore the therapeutic potential of modulating eosinophil activation in diverse inflammatory contexts.

TGF-β is known to be produced by alveolar macrophages and type II airway epithelial cells [18], and it has been implicated in the pathogenesis of pulmonary fibrosis [19]. In the context of asthma, TGF-β levels increase following allergen exposure [20] and are believed to contribute to airway remodeling. Additionally, TGF-β signaling has been extensively studied as a regulator in colitis pathogenesis, particularly through its influence on IL-17 family production [21, 22]. In our study, we have revealed a novel function of TGF-β, shedding light on how it promotes both experimental asthma and colitis. These findings emphasize the significance of TGF-β signaling in eosinophil activation and its impact on inflammatory responses in different disease contexts.

Our results from both in vitro and in vivo experiments indicate that TGF-β drives eosinophil activation. Interestingly, while TGF-β inhibition significantly affected colitis inflammation, it had no notable impact on asthma inflammation in mice. This paradoxical phenotype warrants further investigation to elucidate the underlying reasons. Firstly, to further clarify the origin of TGF-β, we reviewed published articles utilizing single-cell analyses of asthma [23] and colitis tissues [24]. Single-cell sequencing indicates that the primary source of TGF-β varies between these models: B cells in asthmatic lungs and endothelial cells in colitis tissues (Fig. S7). Additionally, TGF-β is usually secreted and stored in a latent form, with activation being crucial for its regulatory function. Therefore, transcriptional expression levels may not accurately reflect active TGF-β levels, highlighting the need for further experiments to detect active TGF-β for more reliable data.

An alternative possibility involves granule proteins. Previous research has highlighted the crucial role of eosinophil-derived granule proteins in asthma development [9], with their levels correlating with disease severity [10, 25]. However, EA Jacobsen et al. showed that Epx and Mbp deficiency did not improve asthmatic pathology in I5/hE2 mice, attributing this discrepancy to potential differences between human and mouse eosinophils, particularly the lack of extensive degranulation in allergic mouse models [26]. As a result, we sought to identify a mouse model where peroxidase may exert pathological effects. Based on reported research, we identified that eosinophils are involved in the DSS-induced colitis mouse model [5, 27]. The observed pattern of Epx immunofluorescence of both HDM and DSS model (Fig. 4D and Fig. 5F) is consistent with the notion that eosinophil degranulation may not exert a discernible influence on the pathology of asthma in murine models. Consequently, the HDM model may not be deemed an entirely ideal murine model for comprehensively investigating the role of eosinophils in asthma. Future investigations should consider exploring additional models to gain a more nuanced understanding of eosinophil involvement in asthmatic pathophysiology.

The precise definition of “eosinophil activation” remains heterogeneous among studies [28], and the specific cytokines responsible for this process are not yet fully elucidated. Our study aimed to clarify eosinophil activation, particularly focusing on the role of TGF-β. We demonstrated that TGF-β is critical for eosinophil activation, as indicated by the increased expression of the activation marker CD101 [7]. Furthermore, TGF-β was shown to promote eosinophil peroxidase (EPX) secretion, which aligns with clinical studies suggesting that EPX may serve as a biomarker for eosinophil activation in sputum samples. The identification of TGF-β as a key factor in eosinophil activation provides valuable insights into the underlying mechanisms driving eosinophil-mediated inflammatory responses. During our study, we made an interesting observation that IL-33 induces an alternative activation of eosinophils, characterized by increased expression of Th2 cytokines such as IL-4, IL-5, and IL-13 (data not shown). Importantly, TGF-β did not affect eosinophil expression of these Th2 cytokines (data not shown). Based on our previous study, activated eosinophils (marked by CD101^+^) promoted lipopolysaccharide (LPS)-induced lung inflammation and injury [8]. However, we observed no reduction in airway inflammation when TGF-β signaling and eosinophil activation were inhibited in HDM-LPS overlap mice (Fig. S8). This finding suggests that multiple pathways may contribute to eosinophil activation, highlighting the need for further research into the heterogeneity of eosinophil activation and its implications for eosinophil-related diseases.

Accumulating evidence highlights the crucial role of Eos in regulating intestinal diseases [11, 28]. Eos are known to significantly impact intestinal inflammation and repair, and their impact on the intestinal immune microenvironment has been extensively discussed. For instance, JC Masterson and colleagues have reported that Eos attenuate DSS-induced colitis by secreting anti-inflammatory mediators [5]. Additionally, some studies suggest that Eos modulate inflammation by regulating the Th1/Th2 balance [29]. However, the role of Eos in colitis remains a subject of debate. Levy et al. observed increased Eos numbers and degranulation in the colon tissue of colitis patients [27], possibly due to CCL11 oversecretion by colon macrophages and epithelial cells [30]. To reconcile this discrepancy, Gurtner and colleagues identified different Eos subtypes in the colon [31], including active eosinophils expressing CD80 and PD-L1, which differ from the active eosinophils (CD101^+^CD62L^−^) observed in lung tissue [7]. These findings indicate that active Eos may participate in inflammation, their functional characteristics may vary depending on the tissue context. A detailed comparison of eosinophils in the lung and colon is warranted to better understand their diverse functions. In our study, we found that active Eos promoted experimental colitis, and inhibiting TGF-β-induced Eos activation attenuated the inflammatory response in the intestine. However, the mechanistic basis of how Eos activation regulates colitis remains unclear, necessitating further investigation in subsequent studies.

In conclusion, our study sheds light on the role of Eos activation in TGF-β-driven inflammatory responses and identifies a potential therapeutic target for Eos-associated inflammation. Understanding the mechanisms underlying Eos activation and its impact on different inflammatory conditions may lead to novel therapeutic strategies for managing eosinophil-related diseases, such as asthma and colitis. Further investigations in this area will advance our knowledge of Eos biology and contribute to the development of targeted therapies aimed at modulating Eos activation in various inflammatory contexts.

### Supplementary information

supplement figure legends

Uncropped blots

## Data Availability

RNAseq data was performed and analyzed from our previous research [8]. Raw data had been uploaded in NCBI under BioProject ID: PRJNA479696.
