# Supplementary material for: TGF-β signaling promotes eosinophil activation in inflammatory responses
Source: Cell Death Dis. 2024 Aug 30;15(8):637. doi: 10.1038/s41419-024-07029-2 (PMC11364686; doi:10.1038/s41419-024-07029-2)

TGF-β signaling promotes eosinophil activation in inflammatory responses

Chen Zhu#,[1] Qingyu Weng#,[1] Shenwei Gao#,[1] Fei Li,[1] Zhouyang Li,[1] Yinfang Wu,[1] Yanping Wu,[1] Miao Li,[1] Yun Zhao, [1] Yinling Han, [1] Weina Lu,[2] Zhongnan Qin,[1] Fangyi Yu,[1] Jiafei Lou,[1] Songmin Ying,[1] Huahao Shen*,[1,3] Zhihua Chen*,[1] Wen Li*[1]

1 Key Laboratory of Respiratory Disease of Zhejiang Province, Department of Respiratory and Critical Care Medicine, The Second Affiliated Hospital of Zhejiang University School of Medicine, Hangzhou, Zhejiang, 310009, China

2 Surgery Intensive Care Unit, The Second Affiliated Hospital of Zhejiang University School of Medicine, Hangzhou, Zhejiang, 310009, China

3 State Key Lab for Respiratory Diseases, Guangzhou, Guangdong, 510120, China.

Supplementary Figure legend

Fig. S1 Purity of isolated Eos from NJ1638 mice.

Fig. S2. Representative FACS gating strategies of Eos. (A) Human peripheral blood. (B) Lung tissue of HDM model. (C) Colon tissue of DSS model.

Fig. S3. IL-12 cannot induce the expression of CD101 in eosinophils. CD101 expression (as mean fluorescence intensity) of eosinophils by FACS. Sample size is indicated as individual plots in column graphs. Data are triplicate by individual experiments. *, $p<0.05$

Fig. S4. TGF-β cannot induce the expression of *Il4* and *Il13* in eosinophils. Left, relative mRNA expression of *Il-4*, Right, relative mRNA expression of *Il-13*. ns, not significant. Sample size is indicated as individual plots in column graphs. Data are triplicate by individual experiments.

Fig. S5. TGF-β also activated bone marrow derived Eos. (A) Mean fluorescence intensity of CD101 in Eos. (B) Decrease EPX in Eos of Tgfbr2 specific knockout mice. Left, relative mRNA expression of *Epx*. Right, representative images of immunofluorescence of Eos. Sample size is indicated as individual plots in column graphs. Data are triplicate by individual experiments. **, $p < 0.01$; ****, $p < 0.0001$.

Fig. S6. Airway inflammation in a chronic HDM model. (A) Scheme of model establishment. (B) Cellularity of total cells in BALF. (C) Percentage of Eos in lung homogenates. Sample size is indicated as individual plots in column graphs. Data are triplicate by individual experiments. ns, not significant.

Fig. S7. Single-cell analyses of asthma and colitis tissues. (A) UMAP plot of depicting

the subsets of asthmatic lung tissue, and expression analysis of Tgfb1 in single cell data. (B) UMAP plot of depicting the subsets of colitis colon tissue, and expression analysis of Tgfb1 in single cell data.

Fig. S8. Airway inflammation in HDM/LPS overlap model. (A) Cellularity of cells in BALF. Left, total cells count in BALF. Middle, percentage of neutrophils in BALF. Right, percentage of Eos in BALF. Sample size is indicated as individual plots in column graphs. Data are triplicate by individual experiments. ns, not significant.

Fig S1.

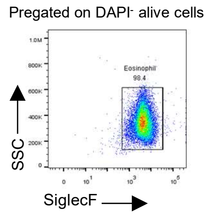

Fig S2.

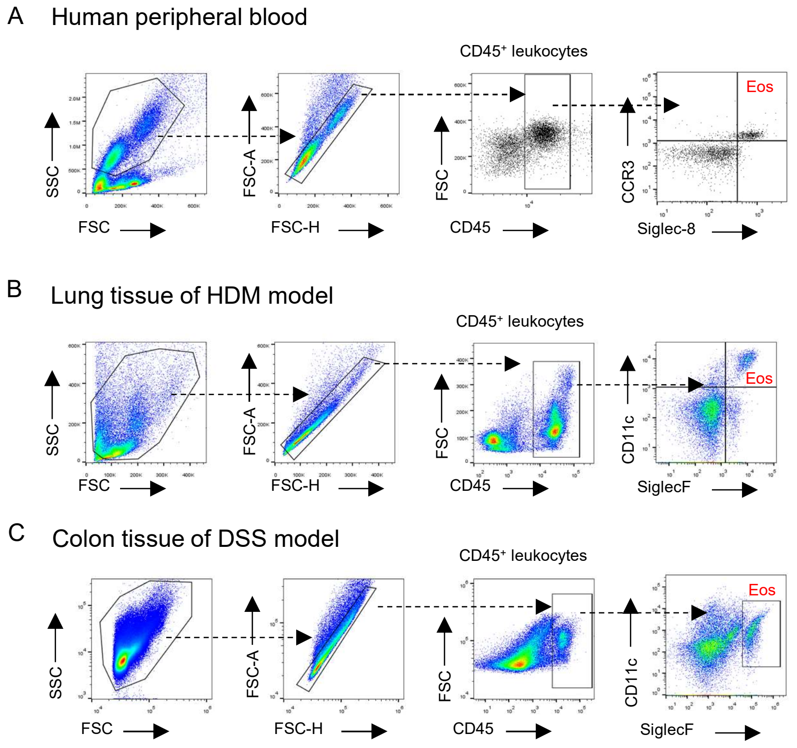

Fig S3.

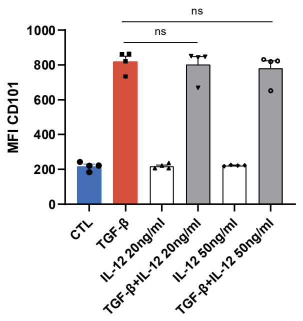

Fig S4.

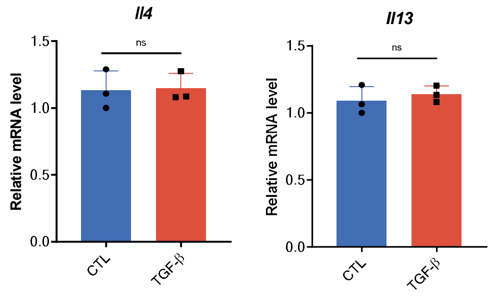

Fig S5.

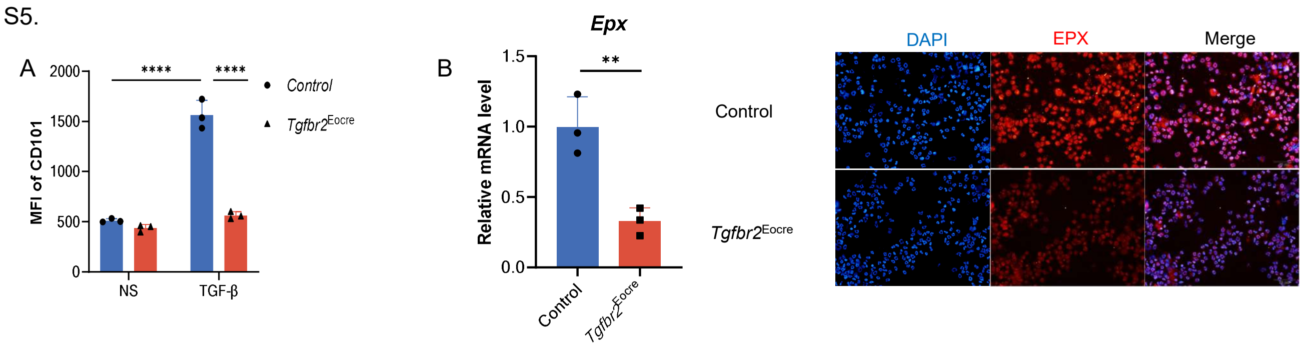

Fig S6.

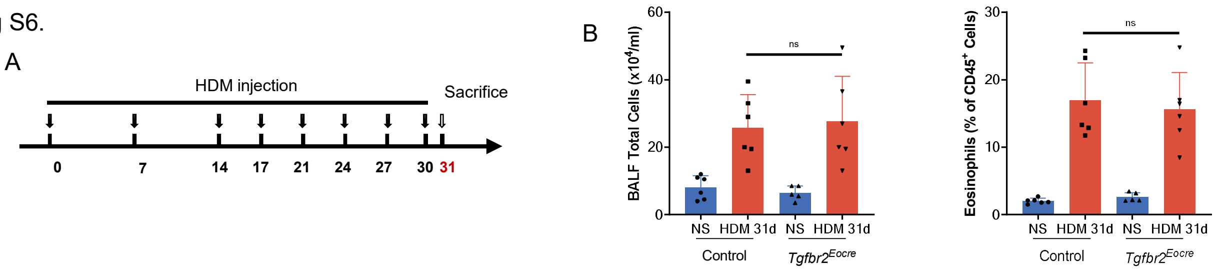

Fig S7.

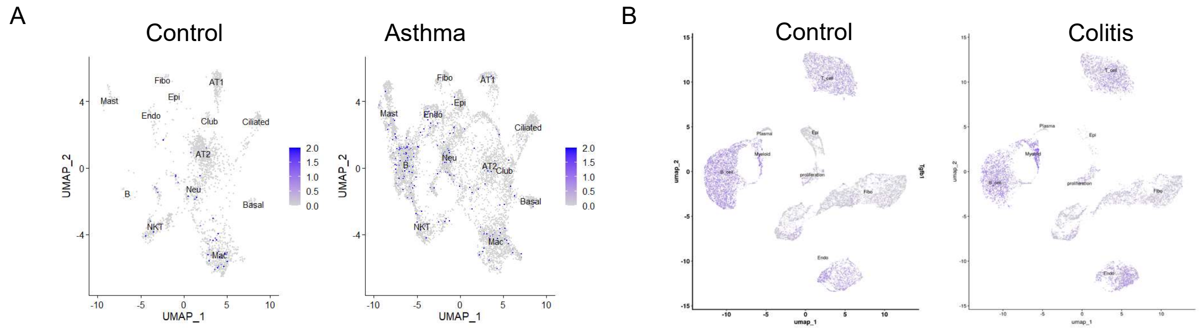

Fig S8.

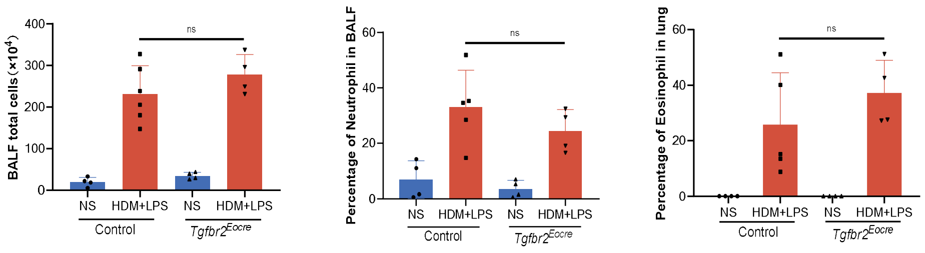

Supplement: Supplementary file 1 — supplement figure legends [file 41419_2024_7029_MOESM1_ESM.pdf]
