# Supplementary material for: TGF-β signaling promotes eosinophil activation in inflammatory responses
Source: Cell Death Dis. 2024 Aug 30;15(8):637. doi: 10.1038/s41419-024-07029-2 (PMC11364686; doi:10.1038/s41419-024-07029-2)
Supplement: Supplementary file 2 — Uncropped blots [file 41419_2024_7029_MOESM2_ESM.pptx]

## Slide 1
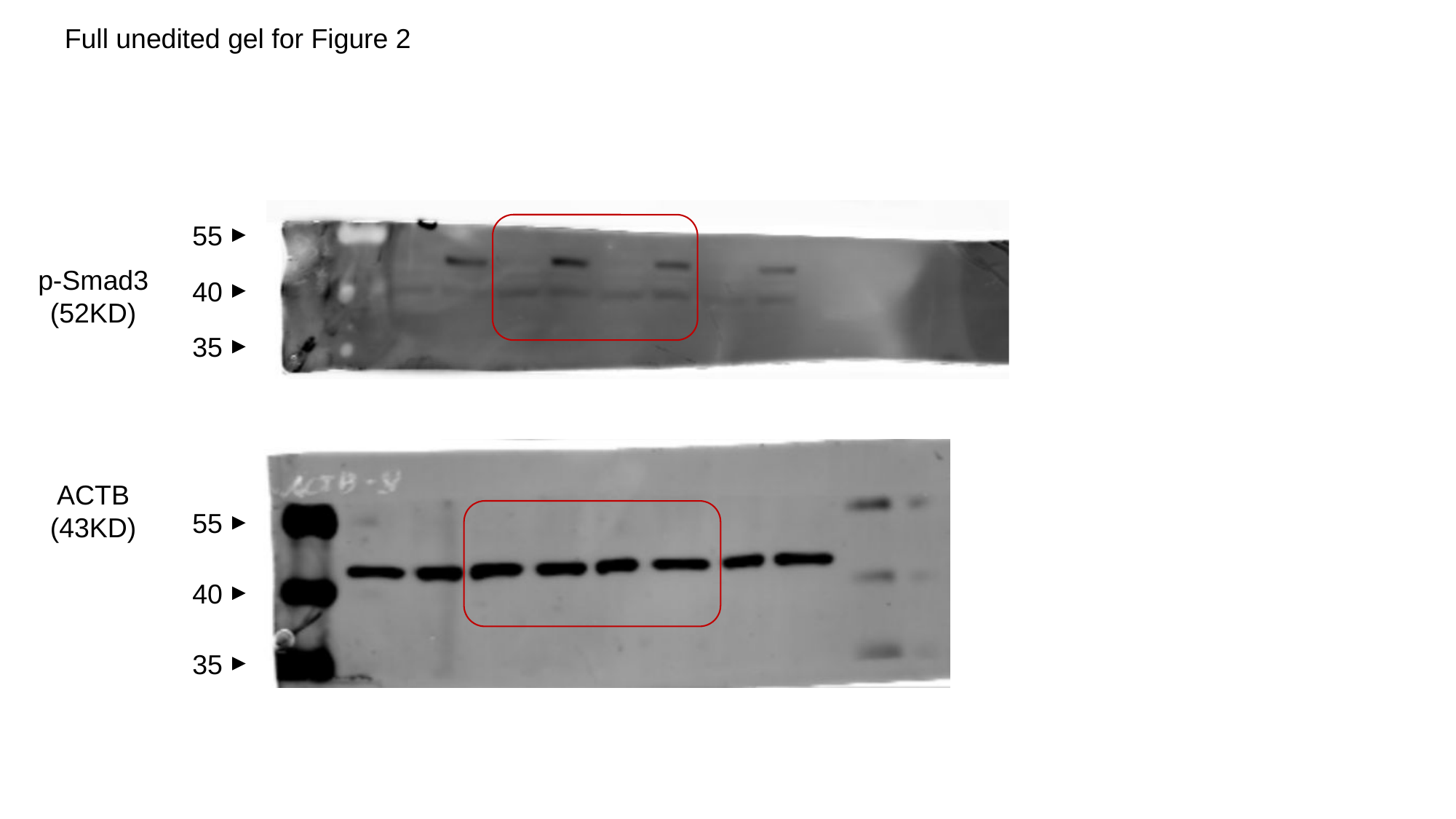

Full unedited gel for Figure 2
55
p-Smad3
(52KD)
40
35
ACTB
(43KD)
55
40
35

## Slide 2
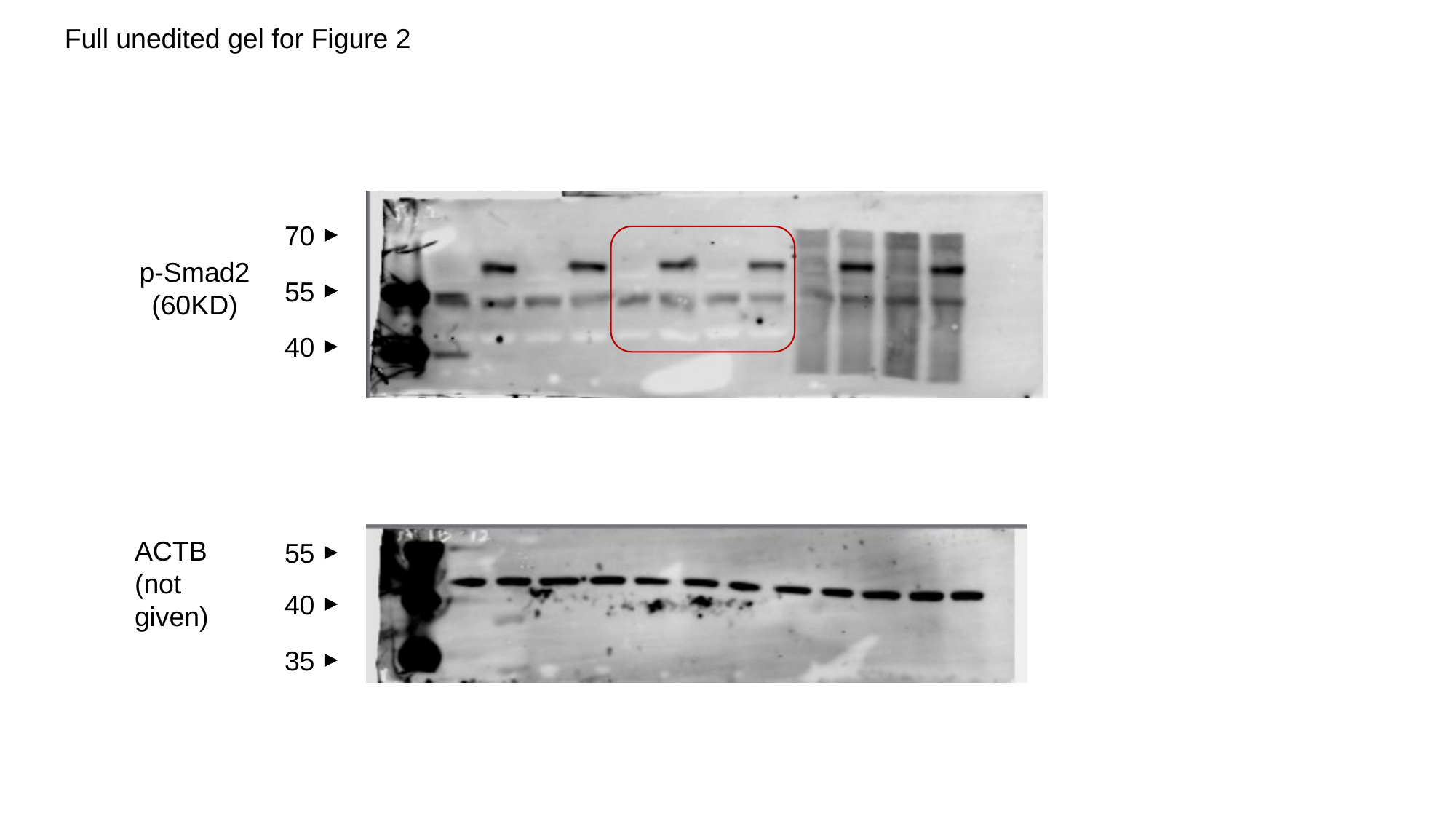

Full unedited gel for Figure 2
70
p-Smad2
(60KD)
55
40
ACTB
(not given)
55
40
35

## Slide 3
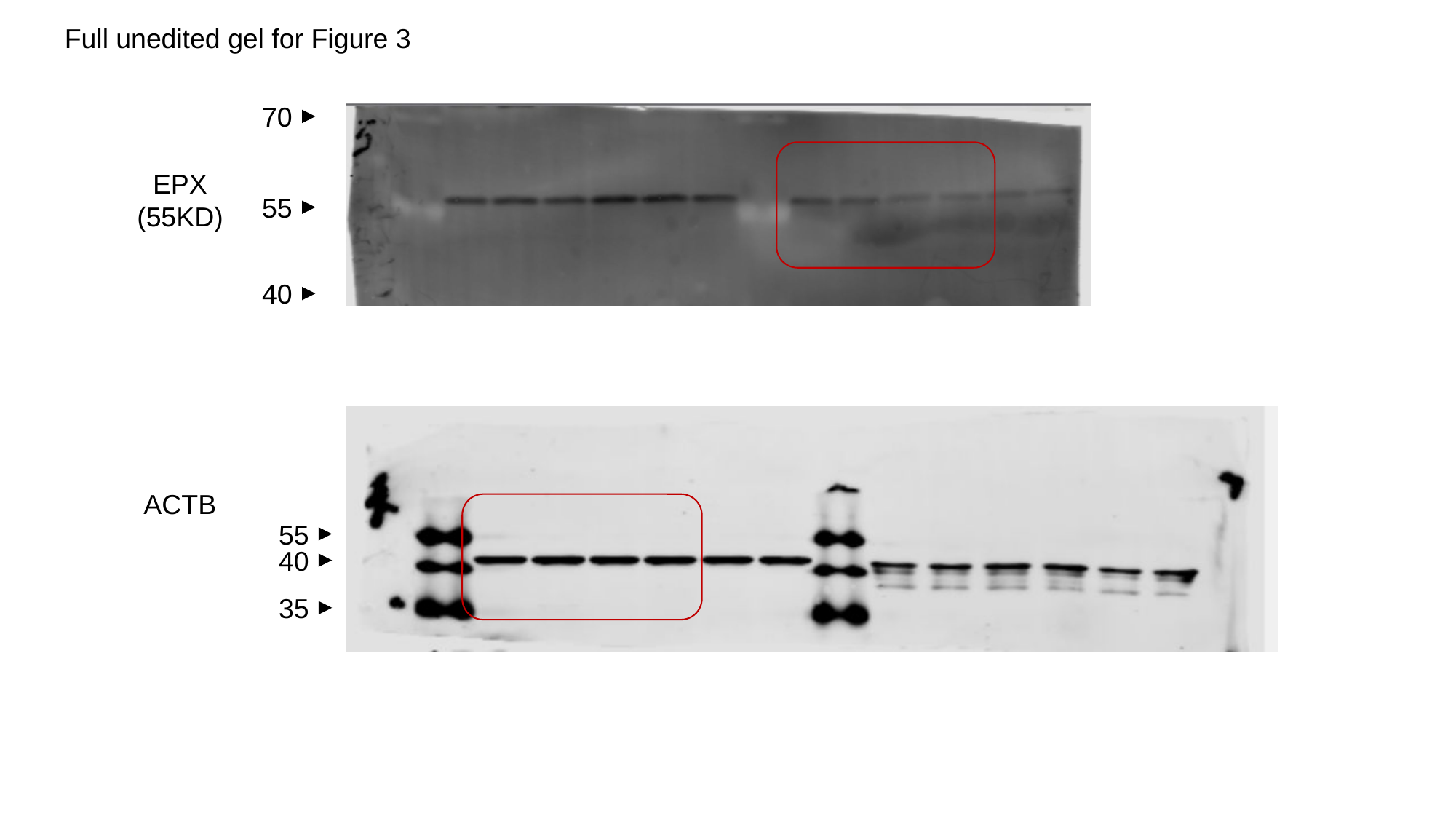

Full unedited gel for Figure 3
70
EPX
(55KD)
55
40
ACTB
55
40
35
